# Congenital CMV and Hearing Loss—How Does it Happen and How to Prevent it

**DOI:** 10.1002/rmv.70156

**Published:** 2026-04-25

**Authors:** Karen B. Fowler

**Affiliations:** ^1^ Department of Pediatrics Heersink School of Medicine University of Alabama at Birmingham Birmingham Alabama USA

**Keywords:** congenital CMV infection, cytomegalovirus, sensorineural hearing loss

## Abstract

Congenital cytomegalovirus (cCMV) is found worldwide and significantly contributes to permanent childhood hearing loss. CMV has been known to cause sensorineural hearing loss (SNHL) for more than half a century, and CMV‐related hearing loss has consistently been present in all childhood populations where infants with cCMV have been identified and followed in the first years of life. CMV‐related hearing loss has a variable onset, with some cases of hearing loss occurring at birth and others developing within the first 5 years of life. Further deterioration of hearing loss may occur in children with CMV‐related hearing loss. In contrast, other children experience improvements or fluctuating hearing loss. SNHL due to CMV is likely to involve both direct viral‐mediated damage that occurs when the virus infects cells within the cochlea and immune or inflammatory responses in the inner ear. The studies in animal models and human temporal bones support that virus infection and host inflammatory responses may lead to both virus‐mediated and virus and host‐derived damage to the auditory system. As more children are being tested for CMV by newborn screening, SNHL is being identified more often. Current recommendations are for valganciclovir treatment in children with cCMV infection and SNHL; however, research gaps exist regarding the long‐term effectiveness of valganciclovir. Without a licenced CMV vaccine, CMV behavioural preventative measures that minimise maternal saliva and urine exposures from young children is the only primary intervention available to reduce CMV infections and, therefore, CMV‐related SNHL. Lack of CMV awareness and knowledge of the CMV disease burden in the population limits this approach, and more CMV education is needed. Data gaps exist in estimating the public health burden and lifetime economic burden of CMV. Without this data, it is not possible to accurately evaluate the cost‐effectiveness of any CMV intervention or prevention strategy. Even after decades of attempting to identify which children with cCMV will have SNHL, further progression of loss, or late‐onset loss, the need remains to identify the children with cCMV at increased risk of SNHL to provide timely detection and intervention for possible hearing loss.

**Abbreviations**
AAP: American Academy of Paediatrics
ABR: Auditory brainstem response
cCMV: congenital CMV
CI: confidence interval
DALY: disability adjusted life year
DBS: Dried blood spot
ECCI: European Congenital Cytomegalovirus Infection Initiative
HIC: high‐income countries
HIG: Hyperimmune immunoglobulin
LMIC: low‐to middle‐income countries
MRI: Magnetic resonance imaging
OAE: otoacoustic emissions
OR: odds ratio
RCT: Randomised controlled trials
SNHL: sensorineural hearing loss

## 1 Introduction

Congenital cytomegalovirus (cCMV) is found worldwide. It contributes to thousands of children each year being born with or developing permanent disabilities such as hearing loss, cognitive impairment, vision loss, and/or cerebral palsy. cCMV‐related disease is more common among infants and young children than other, more recognised diseases such as Down syndrome, foetal alcohol syndrome, or spina bifida [1]. cCMV significantly contributes to permanent childhood hearing loss, with CMV‐related hearing loss being second only to genetic causes [2, 3].

Human CMV is a ubiquitous herpesvirus that spreads through bodily fluids such as saliva, urine, blood, vaginal secretions, and semen, and in most populations, CMV seropositivity rates increase with age. CMV may be transmitted during the perinatal period to infants from their mothers either during birth through the genital tract or through breast milk [4]. After early infancy, young children acquire CMV through horizontal contamination from child to child or through environmental contamination, especially in children who attend group childcare centres [4]. Young children excreting the virus may become the source of CMV infection for parents and others in the household or susceptible childcare providers. Another source of CMV infection is intimate contact with oral and genital secretions through sexual activity [4]. For women of childbearing age, the exposures to CMV either through young children or sexual activity, or both, may result in a CMV infection that may be transmitted to their foetus during pregnancy with a resulting cCMV infection in their newborn. Although we know what factors contribute to CMV acquisition among women of childbearing age, we do not know which of the women will deliver a newborn with cCMV. Both women with a primary infection during pregnancy and CMV seroimmune women can deliver a newborn with cCMV infection. In the USA, Canada, Western Europe, and Australia, cCMV occurs in 2–7 per 1000 of all live births [5, 6, 7, 8, 9, 10, 11, 12, 13, 14, 15, 16, 17, 18]. In other parts of the world, such as Latin America, Africa, and certain Asian countries, cCMV rates are even higher, at 10 to 20 per 1000 live births [19, 20, 21, 22, 23]. Overall, low‐to middle‐income countries (LMICs) have a pooled cCMV prevalence of 14.2 per 1000 live births (95% confidence interval (CI), 9.7–20.8 per 1000) versus the high‐income countries (HICs) pooled cCMV prevalence of 3.8 per 1000 live births (95% CI, 2.3–6.6 per 1000) [24]. Risk factors associated with higher cCMV prevalence rates include young maternal age at delivery, lower socioeconomic status, higher maternal CMV rates, and populations with higher HIV prevalence [7, 11, 24]. Additionally, young women caring for young children before and during pregnancy have an increased risk of cCMV in their newborns [25, 26].

Even though cCMV prevalence varies based on the underlying risk factors in the maternal population, once the foetus is infected with CMV, the spectrum of disease ranges from clinically inapparent infection to potentially life‐threatening disease and possibly death in newborns. Approximately 10%–15% of infants with cCMV have clinical abnormalities at birth, including evidence of disseminated disease and/or CNS involvement [11, 24, 27]. Although cCMV seroprevalence differs between HICs and LMICs, rates of pooled symptomatic cCMV are similar (11% vs. 10.4% for HICs and LMICs, respectively) [24]. About 40%–60% of symptomatic infants will suffer from permanent sequelae, with sensorineural hearing loss (SNHL) being the most common, followed by cognitive impairment, retinitis, and cerebral palsy with and without intellectual disability [8, 28, 29, 30, 31, 32]. However, 85%–90% of infants with cCMV will exhibit no clinical manifestations (asymptomatic) during the newborn period. Asymptomatic infants are not without risk of CMV‐related disease, although a smaller percentage will have permanent sequelae due to CMV infection. The most common sequela in children following an asymptomatic cCMV infection is SNHL, with about 15% of asymptomatic infants developing SNHL following infection [2, 8, 30, 31, 33].

The disease burden of children with cCMV, besides SNHL, may include intellectual disability, learning disorders, developmental delay, retinitis, seizure disorders, cerebral palsy, and autism spectrum disorders [34]. Children with cCMV may have mild to moderate developmental delay/disability in childhood and adolescence, with some progressing to permanent disabilities in adulthood [35]. Additionally, cCMV disease contributes to infant mortality, with reported estimates of 4–8 per 1000 liveborn infants with cCMV who will die in early infancy in HICs [36]. However, due to gaps and limitations in the existing data, the true magnitude of the risk is uncertain. Also, data on the total burden of mortality due to cCMV, including foetal deaths and excess deaths in childhood, are unknown. cCMV is often underrecognized, as estimates of the public health impact of cCMV are not available. Only one study in Belgium has assessed the public health impact of cCMV by quantifying the disease burden in terms of disability adjusted life years (DALYs) [37]. The total public health impact was 1976 DALYs (uncertainty interval, 757–4067) in Belgium in 2013, and was higher if foetal loss was included [37].

The economic burden of managing cCMV and its sequelae is expensive [38, 39]. Additionally, hearing loss incurs a significant societal cost. For a child with prelingual deafness, the estimation exceeds $1 million in the USA, double the cost associated with strokes, epilepsy, and rheumatoid arthritis [40]. However, the economic burden of cCMV in the United States is not well understood due to gaps in cCMV disease burden data [35]. An economic study in the UK estimated the cost of cCMV in 2016 to be £732 million (∼$897 million in USA dollars) [41]. A Japanese study estimated the economic impact of cCMV for the 2019 Japanese birth cohort to be approximately ¥27.6 billion (∼$252 million in USA dollars) [42]. The cCMV cost estimates would likely be even higher in countries, including the USA, where more infants with cCMV are born each year than in the UK or Japan.

With the disease burden of cCMV and the associated economic costs of cCMV disease, prevention of CMV is needed. However, prevention of CMV has remained elusive. Although vaccine strategies are promising, currently there are no licenced CMV vaccines to prevent CMV infection in pregnant women. Therefore, the focus on primary prevention of CMV has been on preventing CMV infection in the mother and, thus, avoiding vertical transmission to the foetus and subsequent cCMV disease by adapting behavioural risk reduction and hygiene measures for pregnant women to prevent CMV [43]. This approach relies on knowledge of CMV and its transmission mode, which is hindered by limited CMV awareness and a lack of understanding of the significant CMV disease burden in the population. Secondary prevention of treating a maternal CMV infection during pregnancy and potentially preventing intrauterine transmission to the foetus has had limited success, although studies are ongoing [43].

### 1.1 CMV Hearing Loss Burden

CMV has been known to cause SNHL for more than half a century [44]. Over the decades, CMV‐related hearing loss seems to be consistently present in all childhood populations where infants with cCMV have been identified at birth or have been followed longitudinally in the first years of life [29, 30, 45, 46, 47, 48]. SNHL following cCMV infection does not have a pathognomonic audiometric configuration and varies in severity of loss. The incidence of SNHL in children with cCMV has been reported to be between 12% and 20% [2, 30, 48, 49, 50, 51, 52, 53, 54]. Those with symptomatic infections have a higher risk of developing SNHL, ranging from 40% to 63% [30, 51, 52, 53, 54].

Unilateral and bilateral hearing losses may occur in children with congenital CMV infection, with loss varying from unilateral high‐frequency losses (4–8 kHz frequencies only) to profound bilateral losses [30, 48, 50, 51, 52, 53, 54]. Among the children with CMV‐related SNHL, those with asymptomatic cCMV are significantly more likely to have unilateral loss (56.9%, 95% CI, 41.4%–71.8%) than the children with symptomatic cCMV (28.8%, 95% CI, 22.2%–35.9%) [48]. Also, children with cCMV‐related hearing loss experience bilateral severe to profound hearing loss in both groups at 42.6% (95% CI, 20.2%–66.7%) in children with asymptomatic cCMV and 65.1% (95% CI, 54.2%–75.2%) in those with symptomatic cCMV [48]. Children with CMV‐related bilateral hearing loss may also have asymmetric hearing loss [55]. Asymmetric hearing loss is defined as a difference in hearing loss (> 15 dB thresholds at 500, 1000, and 2000 Hz or > 20 dB at 3000, 4000, and 6000 Hz) in each ear [56]. Asymmetric hearing loss, like unilateral hearing loss, may have negative consequences for speech and language development without interventions [57]. A recent small study has found that children with cCMV and isolated SNHL have greater hearing threshold asymmetry compared to children with an enlarged vestibular aqueduct or a connexin 26 mutation [58]. The study found that when the threshold difference between the two ears was greater than 58.6 dB, it was predictive of cCMV infection [58].

CMV‐related SNHL may be present at birth or later in childhood. The incidence of late‐onset hearing loss ranges between 1.3% and 10.6%, although higher incidences have been reported in children with symptomatic infections [30, 33, 48, 52, 54, 59]. Late‐onset hearing loss occurs later in children with asymptomatic cCMV infections than in children with symptomatic infections [30]. Although by 5 years of age, the risk of late‐onset hearing loss in children with cCMV infection is not greater than in non‐infected children [60]. It has been proposed that children with cCMV and normal hearing at 5 years of age do not require additional audiological follow‐up [54, 61]. However, there is no consensus on the length of audiological follow‐up needed for children with cCMV and normal hearing.

Progression or further deterioration of hearing loss occurs in children with hearing loss [30, 48, 51, 52, 53, 62, 63, 64]. Children with both symptomatic and asymptomatic infections may have progression of their hearing loss, ranging from 17.7% (95% CI, 3.5%–39.4%) in the symptomatic group and 20.3% (95% CI, 5.3%–41.8%) in the asymptomatic group [48]. These children may have hearing loss progression throughout adolescence, so unlike children without hearing loss by 5 years of age, they should continue to have audiological follow‐up throughout childhood and into adolescence [60]. Children with cCMV can also experience improvement in their hearing loss, while others with cCMV may exhibit threshold fluctuations with periods of improvement and worsening [30, 48, 50, 54].

The most common audiometric configuration pattern [65] observed in both the symptomatic and asymptomatic groups of children is a flat (approximately equal degree of loss at all frequencies where the slope between frequencies is not > 10 dB) pattern of loss [30, 61]. The presence of this configuration, along with the recently reported hearing threshold asymmetry in children with isolated SNHL, may provide a way to distinguish CMV‐related hearing loss from other types of SNHL for diagnosing older children. Additionally, findings of a cross‐sectional study have identified clinical risk factors for SNHL at birth in children with cCMV. These included petechiae at birth, periventricular cysts on MRI, or maternal seroconversion in the first trimester [66]. These data may provide further insights into the pathogenesis of SNHL due to cCMV.

Although much is known about CMV‐related SNHL, the underlying mechanisms of its pathogenesis are not fully explained [67, 68, 69]. SNHL due to CMV likely involves both direct viral‐mediated damage that occurs when the virus infects cells within the cochlea and immune or inflammatory responses in the inner ear. Few human studies exist due to the challenges in studying inner ear damage and resulting SNHL. Some of the challenges include quantifying viral loads in the inner ear, host heterogeneity in the host antiviral responses to foetal infection, and the limited availability of post‐mortem tissue from the inner ear [69]. However, histologic analysis of temporal bones from a small number of foetuses and newborns with cCMV has identified primary targets of the virus within the inner ear [70, 71, 72, 73, 74, 75]. These studies of histologic autopsies identified the presence of cytomegalic cells in the different compartments of the inner ear, including the vestibular part (semicircular canals and otolith organs), demonstrating the viral tropism of CMV in the inner ear [71, 72, 74, 76, 77, 78]. CMV can be found in the endolymphatic structures, in the stria vascularis, Reissner's membrane, and the labyrinth's dark cells [67]. Species barriers limit the use of experimental animal models. But animal models, including guinea pig and murine models, have been used for studying the inner ear following foetal infection, providing insights into CMV‐related hearing loss. Studies in guinea pigs have been informative, identifying CMV labyrinthitis and hearing loss [79, 80]. The work in guinea pig models has shown that viral gene expression was needed for damage to the inner ear, but an intact host immune response was also required [79, 81, 82, 83]. Additional studies have shown that murine CMV infections in newborn mice have haematogenous spread of virus to the inner ear, inflammatory responses, and hearing loss [84, 85, 86]. The studies in animal models and human temporal bones support that virus infection and host inflammatory responses may lead to both virus‐mediated and virus and host‐derived damage to the auditory system.

Some of the clinical patterns seen, such as a flat configuration, may be indicative of damage to the inner ear throughout the cochlea [61, 68, 74]. CMV has been found in the inner ear in the perilymphatic fluid of children undergoing later cochlear implantation, demonstrating viral persistence [87]. Potassium recycling in the endolymphatic sector of the inner ear is essential for normal hearing. CMV lesions may disrupt potassium recycling, which could explain the delay or fluctuation of hearing loss observed in CMV‐related hearing loss [74]. Also, host persistent inflammation, rather than viral damage alone, may contribute to progressive and late‐onset hearing loss that is seen in CMV‐related SNHL.

[87] Related to SNHL is increasing evidence that vestibular and balance disorders occur in children with cCMV [88, 89, 90, 91, 92, 93]. Both children with SNHL and those without hearing loss may have vestibular impairments. However, the natural history of cCMV‐associated vestibular deficit is not fully elucidated [69]. The vestibular system includes the three semicircular canals and the two otolith organs, the utricle and saccule, in each inner ear, and the central vestibular complex of the spinal cord, cerebellum, and ocular‐motor cranial nerve. An intact vestibular system is needed for gaze stability and postural control [69]. Recent data suggest that vestibular involvement related to CMV, like SNHL, can differ by timing of onset, degree of deficit, and continued deterioration during childhood [88, 89, 93]. The otolith and canal function can be affected in vestibular dysfunction, and vestibular impairment may fluctuate in children with cCMV during childhood [88]. The impairment of the otolith and canal in vestibular function may be a predictor of delayed‐onset hearing loss [90]. Also, children with cCMV may have normal hearing but vestibular deficits, suggesting that the vestibular part of the inner ear may be impaired more often than the cochlea [90, 92, 93]. Currently, there are no standardised vestibular monitoring protocols for children with cCMV. However, the ECCI and the American Academy of Audiology have recommended including vestibular assessments in the management of children with cCMV [94, 95, 96].

### 1.2 Identifying CMV and CMV‐Related SNHL

cCMV is often missed in part because CMV infection can only be identified from urine, saliva, or blood samples collected within the first 21 days of life [27]. Without CMV testing at birth, most children with cCMV go undetected and are not monitored for SNHL [97]. Early cCMV studies in the 1970s and 1980s in the USA, Canada, Sweden, and the UK were research‐focused and identified and described cCMV and CMV‐related hearing loss as part of their investigations [13, 28, 29, 30, 45, 46, 47, 98, 99]. At that time, newborns were not routinely tested for CMV in the nursery, unless they presented with clinically apparent signs and symptoms of cCMV, where they would be tested for CMV on an individual basis. They were often missed, especially if they had nonspecific or milder symptoms [100, 101]. This approach also missed asymptomatic infants, and if they had hearing loss, it was likely not known that CMV was the aetiology of the hearing loss. Before the 1990s in the USA, risk criteria‐based newborn hearing screening, which included CMV, was used to identify congenital hearing loss. Even with this approach, CMV was often not identified [102].

The global transition from a risk‐criteria‐based approach (before the 1990s) to universal newborn hearing screening has led to the widespread adoption of auditory brainstem response (ABR) and/or otoacoustic emissions (OAE) testing in nurseries to identify hearing loss in all newborns. CMV has not been routinely identified by this approach either. Still, recognising that CMV contributes to SNHL, several studies reported that CMV was being identified among those who did not pass their newborn hearing screen [103, 104, 105]. At the same time, CMV polymerase chain reaction (PCR) testing methodology of urine, saliva, or DBS made it possible for less labour‐intensive and rapid testing of CMV [17, 103, 106, 107]. The combination of universal newborn hearing screening, improved CMV PCR testing, and parent advocacy in the USA, Canada, and elsewhere has led to more hospital systems and states in the USA implementing targeted CMV screening, which involves testing a newborn for CMV following a failed newborn hearing screen. Utah was the first state in the USA to implement targeted CMV screening in 2013 [108]. And other states and hospitals have followed both in the USA and worldwide [109, 110, 111, 112, 113].

Although targeted CMV screening identifies CMV, it also misses about 40% of CMV‐related hearing loss [97]. Other studies have evaluated expanded CMV screening, which includes additional factors beyond the targeted approach. Some other factors considered are suspected primary CMV maternal infection, maternal HIV infection, thrombocytopaenia, and growth impairments [114, 115, 116]. An expanded cCMV screening approach identifies more infected infants; however, the number of infants this approach misses is unknown.

Ideally, universal CMV screening would identify all CMV infections, and those infected could be followed for more intensive audiological follow‐up to identify SNHL [11, 15, 117, 118, 119]. In the USA, Minnesota and Connecticut have initiated universal CMV screening, and in Canada, Ontario and Saskatchewan have implemented universal CMV screening [18, 120]. However, since most children with cCMV will not have CMV‐related SNHL, the intensive audiological follow‐up can be both burdensome and expensive for families and health care, and universal CMV screening is not being rapidly adopted. Additional challenges exist, as the DBS methodology used for the current universal programs has low sensitivity, which means it misses infants with cCMV infection [17, 18, 121]. Saliva PCR has high sensitivity, but in some populations, saliva may be contaminated by breast milk, leading to false positives; however, this is not the case in all populations [122]. Urine PCR also has high sensitivity, but collecting urine from newborns can be labour‐intensive and costly. Other methods have been explored, such as umbilical cords in Japan [123] and filter paper for urine [14, 124]. Recently, PCR testing of pooled saliva has been considered for universal screening [125, 126]. This could be an efficient and cost‐saving approach to universal CMV screening, especially in resource‐poor areas. Overall, regardless of the specimen type selected, urine confirmation is crucial, especially before initiating treatment with antivirals for cCMV [27].

Another approach adopted by some EU countries is to focus on performing CMV serology in the first trimester of pregnancy to diagnose primary maternal infection, and to test their infants when suspected or confirmed primary CMV infection occurs during pregnancy [94]. For seronegative women, the European Congenital Cytomegalovirus Infection Initiative (ECCI) consensus recommendations have endorsed that they continue to be tested every 4 weeks until 14–16 weeks, to identify any primary infections that may occur. This approach is based on data that has shown the increased risk of long‐term sequelae in a foetus following a maternal primary infection in the first trimester [127]. The ECCI recommendations also advocate testing for cCMV in any infant suspected of hearing loss at birth.

### 1.3 CMV Prevention Strategies

Primary prevention of maternal infections, and therefore cCMV and related hearing loss, could be achieved by vaccination [94]. A successful vaccine could significantly reduce CMV‐related SNHL, although a licenced CMV vaccine is currently unavailable [128]. However, several vaccines are currently in clinical trials, with one anticipating completing a Phase III trial soon [128]. The mRNA (mRNA 1647, a glycoprotein B and pentameric glycoprotein complex platform) vaccine by Moderna is completing a Phase III trial (NCT05085366) evaluating the efficacy, safety, and immunogenicity in 16–40 year olds and anticipates results by 2026. Another Phase I/IIa trial (NCT05575492) by Moderna is recruiting for the same vaccine candidate in CMV‐negative and CMV‐positive 9–15‐year‐olds and anticipates results in 2027. Also, GlaxoSmithKline has completed enrolment on a Phase I/II trial (NCT05089630) in healthy 18–50‐year‐old adults using a recombinant protein and adjuvant, with results anticipated in 2029.

In place of a licenced vaccine, CMV behavioural preventative measures that minimise maternal saliva and urine exposures from young children are an approach to lower cCMV infections and CMV‐related SNHL. Simple preventative messages include avoiding contact with the saliva and urine of young children: (1) by not sharing food, drinks, cups, utensils, and pacifiers; (2) not kissing on or near a child's mouth; and (3) handwashing after feeding or caring for infants and young children. Education about these behavioural measures has been demonstrated to be acceptable to pregnant women [129, 130, 131]. It has been successfully shown that providing education about increasing preventive measures reduces the risk of seroconversion in CMV‐seronegative women [132, 133, 134]. There is less direct evidence for women who are CMV seropositive before pregnancy, both whether they have exposures to young children or would benefit from CMV behavioural preventative measures. In some populations, CMV seropositivity in women has not been related to exposure to young children [135, 136]. But data from Brazil and the USA indicate that in some populations, CMV seropositive women do have frequent exposures to young children [25, 26 ], so it is likely all women would benefit irrespective of their CMV serostatus [94].

Valaciclovir and hyperimmune immunoglobulin (HIG) have been considered potential options for reducing transplacental transmission and preventing foetal infection as well as subsequent SNHL. A randomised controlled trial (RCT) of valaciclovir of 90 pregnant women who had acquired a primary infection during the periconceptional period or the first trimester were randomised and 11% of the valaciclovir group versus 30% of the placebo group tested positive for CMV on amniocentesis at 21–22 weeks gestation after 6 weeks of treatment demonstrating a decrease in vertical transmission (odds ratio (OR), 0.29; 95% CI, 0.09–0.9, *p* = 0.027) [137]. A case‐control study involving 65 women with a CMV primary infection early in pregnancy who received valaciclovir and 65 matched controls who did not receive treatment found that foetal infection was lower in the treatment group (12%) compared to the untreated group (29%, *p* = 0.029) [138]. Similar data from a retrospective multicenter study found a reduction in the rate of maternal‐foetal CMV transmission following the treatment with valaciclovir (OR, 0.40, 95% CI, 0.18–0.90; *p* = 0.029) [137, 138, 139]. If valaciclovir could be administered soon after a maternal CMV infection, during the periconceptional period or first trimester of gestation, this could result in a possible reduction in cCMV and CMV‐related SNHL, primarily because early trimester maternal CMV infections have been associated with late‐onset SNHL in children with cCMV [140].

However, two RCTs of HIG as an agent to prevent maternal CMV infection and subsequent foetal transmission did not demonstrate a decrease in foetal infection [141, 142]. A randomised placebo‐controlled double‐blind study of 123 pregnant women with primary CMV infections reported the rate of cCMV was 30% in the HIG group and 44% in the placebo group (*p* = 0.13) and did not significantly reduce the rate of intrauterine transmission in women with primary CMV infection [141]. A second, larger randomised controlled trial examined 394 women with primary CMV infections and also did not find a difference in foetal transmission between the groups, with 22.7% of women in the HIG group versus 19.4% in the placebo group having intrauterine transmission of CMV [142]. However, a nonrandomised study of a higher HIG dose administered earlier in pregnancy has found that foetal transmission was significantly reduced [143]. Due to conflicting data, HIG is not recommended for use outside of a research setting [144].

For the infant with cCMV diagnosed by urine, saliva, or DBS by PCR within the first 21 days of life, the infant with signs or symptoms of cCMV may be treated with antivirals, such as valganciclovir or ganciclovir. An RCT of ganciclovir in symptomatic newborns found some benefit for stabilising hearing and improved neurodevelopmental scores at 24 months [145]. The RCT of valganciclovir, which compared 6 weeks to 6 months of treatment in newborns, found greater efficacy for 6 months of treatment [146]. An additional non‐randomised trial for infants with isolated SNHL, starting 6 weeks of valganciclovir treatment as late as 13 weeks of age, showed stable hearing at 20 months of age. However, another RCT in children with SNHL, aged 1 month to 4 years, given 6 weeks of valganciclovir, did not find improvement in their hearing 6 months later [147]. Based on these findings, recommendations for treatment of the newborn with cCMV infection vary on timing and length of treatments depending on the severity of symptoms in the newborn at birth. In the US, the 2024 American Academy of Paediatrics (AAP) Redbook recommends for those infants with moderately to severe cCMV disease receive 6 months valganciclovir and for infants with no clinically apparent signs and only isolated SNHL receive 6 weeks of valganciclovir, with those with mild and transient signs and symptoms to be considered on a case‐by‐case consultation with paediatric infectious diseases experts [147]. Infants with normal hearing and no apparent signs of cCMV disease are not recommended for treatment outside of a research study. The ECCI consensus recommendations for cCMV are slightly different from the AAP guidelines [94]. ECCI guidelines recommend 6 months of valganciclovir for any child with CNS involvement, including hearing loss, and 6 weeks or more for infants with no CNS involvement, such as those with isolated hepatitis or thrombocytopaenia [94]. The ECCI guidelines also recommend joint decision‐making with parents regarding treatment and the benefits versus risks associated with it.

With the current recommendations for antiviral treatment of cCMV infections, it will be essential to recognise that research gaps still exist regarding the long‐term effectiveness of antiviral therapy and SNHL. Recent data indicate that children with cCMV and isolated SNHL, whether congenital‐onset or late‐onset loss, experience the poorer‐hearing ear worsening earlier and more quickly than the other ear [64, 148]. This suggests that evaluating both ears versus a ‘better ear’ approach in future efficacy assessments of antiviral treatments should be considered. Mixed data exist on whether antiviral treatment preserves hearing beyond the treatment period. Several studies have reported that the progression of hearing loss continues after the antiviral treatment period ends [149, 150]. No long‐term data exist on whether valganciclovir decreases the need for hearing aids or cochlear implants, or whether spoken language measures are improved in children treated with valganciclovir.

## 2 Conclusion

Although much is known about CMV epidemiology and outcomes following cCMV infections, data gaps remain. DALYs and the public health burden of cCMV in both HIC and LMIC populations are not known. Understanding and predicting the neurodevelopmental disabilities that children with cCMV may experience, especially those without clinically apparent disease at birth, is needed. Data gaps also exist for determining the lifetime economic burden of cCMV. Without understanding the whole lifetime economic burden, accurately assessing the cost‐effectiveness of any primary and secondary prevention efforts is not possible. As more CMV screening occurs in the newborn population, more children will be identified with cCMV infections, leading to increased CMV awareness in the population and greater knowledge about the availability of primary and secondary prevention strategies to prevent CMV and SNHL.

Even after decades of attempting to identify which children with cCMV will have SNHL, further progression of loss, or late‐onset loss, no biomarkers have been identified to predict all at‐risk children. However, the need remains to identify the children with cCMV at increased risk of SNHL. These children could benefit the most from more frequent audiometric evaluations and limit unnecessary audiological testing for children at low or no risk for CMV‐related SNHL. Identification of children with cCMV who are at increased risk will allow for earlier intervention and treatment strategies for ameliorating the effects of hearing loss on a child's language and development.

## Author Contributions

K.B.F. conceptualised and draughted the manuscript.

## Funding

The author has nothing to report.

## Conflicts of Interest

The author declares no conflicts of interest.

## Data Availability

The author has nothing to report.
